# Multimodal Interaction of MU Plant Landscape Design in Marine Urban Based on Computer Vision Technology

**DOI:** 10.3390/plants12071431

**Published:** 2023-03-23

**Authors:** Jingwen Yuan, Longlong Zhang, Chul-Soo Kim

**Affiliations:** 1 Department of Marine Design Convergence Engineering, Pukyong National University, Busan 48513, Republic of Korea; 2 Department of Industrial Design, Pukyong National University, Busan 48513, Republic of Korea

**Keywords:** marine city, plant landscape, computer vision technology, multimodal interaction, five-view plant system

## Abstract

At present, there is a growing focus on the landscape and environment of ocean cities, with an increasing demand for improved ecological sustainability and aesthetic appeal. With the emergence of computer vision design technologies such as 3D and VR, people have overcome the limitations of traditional paper-based design materials. Through the use of computer software, various forms of expression, such as drawings and animations, can be produced, thereby meeting the diverse demands for showcasing plant landscapes. The purpose of this paper is to study the design of marine urban (MU) botanical landscapes based on computer vision technology (CVT) and multimodal interaction design (MID) theory, so that the design of MU botanical landscape can meet people’s psychological behavior and visual needs, and at the same time enable people to participate in and experience the landscape, so as to better meet people’s needs for viewing, leisure, and entertainment. At the same time, it is hoped that the research of this paper will play a role in promoting the innovation and development of the concept of MU landscape design (LD) in the future, specifically from two levels of theoretical and practical significance. First, at the level of theoretical research: Based on the theory of MID, this paper explores the application of communication and interaction among humans and between humans and the landscape in the design of MU planting, and tries to explore and summarize the content and methods of interactive LD in marine cities with a theoretical basis and research value. The goal is to both enhance the theoretical level of interactive LD, and also provide new reference for future marine city (MC) LD. Second, at the level of practical application: In the field of LD, the new concept of computer vision is introduced to fully understand the visual needs of people and increase the practicality and pleasantness of the MU landscape, hoping to attract more people to come to play and rest. Through the attraction of MU landscapes to tourists, the design and construction of the landscape no longer focus on its appearance, but rather on the participation and experience of people.

## 1. Introduction

Botanical landscaping is the only way to create a beautiful visual effect of nature’s plants, whether in single or group form, so that people can experience the beauty of this effect from the inside out. Plantscaping includes not only the naturally formed plantscape, but also artificial pruning and landscaping to create the perfect plantscape. At present, many gardening companies benefit from the VR industry and have realized its application. Australian architect Beau B. Beza’s team used interactive experience technology to capture and represent the famous botanical landscape of Geelong’s eastern coastal reserve in Australia. At present, most of the research and applications of multi-modal interactive technology in landscape architecture focus on planning and design, and less attention is paid to garden plant landscaping. At the same time, the existing research also lacks the distinction and summary of applying modal interaction technology to landscape architecture. In terms of practical application, it is mainly used to display and communicate the scheme, analyze the design process, teach garden plant landscaping, etc. The use of aspects is also rare.

Many scholars in China have started to discuss what kind of marine urban (MU) landscape environment is the most ideal environment for recreation and play. In the process of MU planting design, the direction of research has shifted to the analysis of low-carbon and ecological landscape environment design, but they are still inseparable from human participation. In China, there are a large number of relevant theoretical studies on interaction design, marine cities, and planted landscape design (LD), but relatively few research institutions and researchers are engaged in multimodal interactive LD research [1]. With the increasing demand for tourism, MU landscaping as an important part of urban planning has become the highlight of urban landscaping, and people’s love for marine nature makes people more inclined to approach marine landscapes. People pay increased attention to the needs of tourists in the construction of marine cities, and they pay attention to the enjoyment and experience of environmental recreation. However, most of the current research on the design of MU planting focuses more on the form and visual impact, while ignoring the communication and interaction among people and between people and scenery [2,3]. Therefore, it is necessary to analyze its importance to plant LD from the perspective of interactive design.

Based on plant design, this paper points out the necessity of plant LD in marine cities by describing the research background of plant LD in marine cities. A design analysis of the four functional areas of a marine city is then carried out with a case study of the coastal plants in Sanya Bay, China. Finally, the design interaction effect of computer vision technology and the multimodal interactive design on the plant landscape of the marine city is analyzed through interviewees’ evaluation of the multimodal interactive design effect based on computer vision technology. We discuss the multi-modal interactive design of marine city plant landscaping through analysis and research on the relationship between interactive design and maritime city plant landscaping. Integrating the concept of multi-modal interaction into plant landscaping maximizes the role of plants in improving the environment to further the sustainable development of ocean cities. The resulting innovative value is mainly reflected in the expansion of design performance tools and display methods, the incremental innovation of traditional thinking methods, and the diversification of landscaping effects. Through the interactive design concept combined with the current situation of local plant landscaping in Sanya City, the corresponding scientific improvement methods and optimization methods are proposed to create a beautiful marine city plant artistic image and local style characteristics, which have particular theoretical research significance and practical value.

## 2. Basic Overview

### 2.1. Concept Introduction

(1) Features of MU plant LD

Coordination: Plant LD should consider the cooperation among individual plants and between individuals and the whole, so that the landscape reflects mutual integration and comfortable and pleasant aesthetics. Determining the similarities and similar characteristics among plants so that the plants match will make people feel coordinated. On the contrary, the contrast effect can be reflected by differences between plants, so that the whole space has contrast, giving people a feeling of excitement and stimulation. This technique is often used in plant landscaping to highlight the theme of a certain area or to draw people’s attention to something [4].

Recreation: Recreation includes play and rest, and the design of MU planting should not only meet the playful nature of visitors, but also provide resting places. Recreational activities need to be realized with the help of a certain external environment, the same activity subject in a different external environment, and different recreational experiences [5].

Ornamental: Ornamental MU plants are plant species with a landscape ornamental function as the main feature and ecological benefits as a supplement. The ornamental nature of MU plants is mainly reflected in the rich colors, different forms, and distinct textures of plants.

(2) CVT

Computer vision technology includes the fields of image recognition, target detection, vision-based simultaneous localization and map building, depth estimation, etc. It is an emerging technology that involves many disciplines, is comprehensive, and has broad application prospects [6].

The binocular depth estimation algorithm captures binocular images after polar alignment correction using a stereo matching algorithm to calculate a reference image. The most commonly used conventional algorithm for binocular vision is the SGBM algorithm (semi-global block matching) known as the semi-global algorithm. The SGBM algorithm first preprocesses the image using the Sobal operator and afterwards calculates the matching cost. The matching cost consists of two parts: the first part is to calculate the BT (Birchfield Tomasi) cost for the preprocessed image and the second part is to calculate the BT cost for the original. The BT cost is developed on the basis of the AD (absolute differences) cost. The AD cost is the absolute value of the pixel gray value difference, and the AD surrogate value of the pixel I_R_(x, y) in the left pixel and the pixel with parallaxd in the right image is:

(1) $$e(x,y,d)=\left|I_{R}(x,y)-I_{T}(x+d,y)\right|$$

The BT cost is similar to the AD cost; the difference is that the BT cost uses the grayscale information of sub-pixels to reduce the effect of noise. the formula of BT cost is shown in Equations (2) and (3), where, represent the pixel intensity functions of the left and right cameras, respectively; x_i_ and y_i_ are the two pixels in the left- and right-eye images for which the dissimilarity is to be calculated; I_L_ represents the linear interpolation of the left pixel function and I_R_ represents the linear interpolation of the right pixel function. The calculation result of Equation (2) represents the dissimilarity between pixel x_i_ and the pixel in the region near y_i_, and the dissimilarity between the final pixels is determined by this value and the minimum value of the dissimilarity with its symmetry:

(2) $$\bar{d}(x_{i},y_{i},I_{L},I_{R})=\underset{y_{i}-\frac{1}{2}\leq y\leq y_{i}+\frac{1}{2}}{\min}\left|I_{L}(x_{i})-\overset{\wedge }{I_{R}}(y)\right|$$

(3) $$d(x_{i},y_{i})=\min\left\{\bar{d}(x_{i},y_{i},I_{L},I_{R},\bar{d}(y_{i},x_{i},I_{R},I_{L})\right\}$$

(3) Multimodal interaction

**Figure 1:**
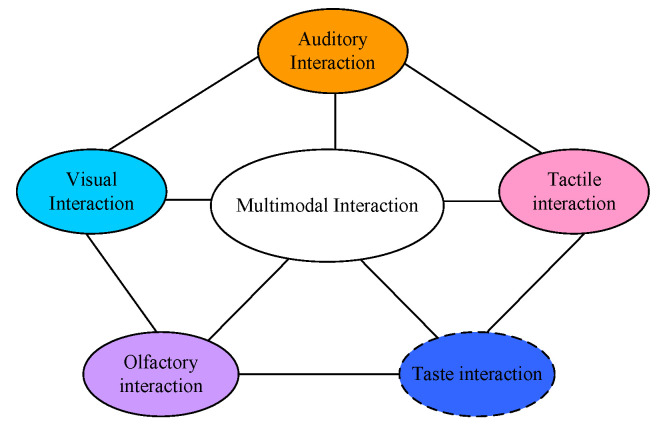
MID concept.

People’s feelings about a landscape are multifaceted, and in the process of experiencing and participating in LD, the landscape stimulates different human senses. In MU interactive LD, human senses are stimulated by different degrees of interactive landscapes, triggering associative effects and prompting a good landscape experience [7]. The MU plant landscape multimodal interactive design concept mainly includes visual, auditory, olfactory, and tactile interaction, while gustatory interaction, although not the main marine correctional plant LD interaction concept, also contributes to multimodal interaction, as shown in Figure 1.

Visual interaction: People feel differences in color, shape, distance, and movement of things through visual perception, which has an impact on their thinking and psychological feelings. In MC interactive LD, visual interaction is used as the main means to attract people to participate in interaction. A clever shape design can mobilize the active participation of visitors. The use of different colors also stimulates people’s vision and brings different feelings to their psychology. The use of colors with higher brightness stimulates the visual senses and generates visual interaction [8].

Auditory interaction: In the interactive LD of marine cities, auditory senses play a large role. Sounds of nature, such as the sound of waves, the chirping of birds, and the sound of wind blowing leaves in the MC, increase the vitality of the MC while visitors can experience physical and psychological relaxation. The sound of various landscape facilities and modern art media as well as people’s conversations stimulates people’s auditory senses and causes different emotional behaviors [9].

Olfactory interaction: Smell is mainly transmitted through scent, and a good smell can elicit a pleasant feeling. In marine cities, the elements that can convey scents are soil, flowers, trees, etc., and artificially added scents. In MC interactive LD, scent is used to influence the viewers’ perceptions and then expand the scope of the interaction between humans and the landscape. Scent can be used to induce people to interact with the landscape; for example, the fragrance of flowers can induce people to come closer to smell them. However, the transmission of smell has certain limitations; generally, the perceptible distance does not exceed 3 m. Based on the different characteristics of each person’s sense of smell, the principle of moderation should be employed during the design [10].

Tactile interaction: touch is the feeling produced by skin receptors in contact with mechanical stimuli, which is one of the most real feelings of human contact with things. In the interactive LD of marine cities, facilities of different materials and plants of different textures are set up to let people get close to the landscape through touch.

### 2.2. Literature Review at Home and Abroad

In October 2018, the Chinese State Council emphasized its commitment to further promoting the development of China’s new generation of artificial intelligence (AI). As a result, many high-tech companies are gradually entering the AI industry. The rapid development of digital media technology in the context of smart city construction has promoted urban information exchange, enhanced connectivity, and promoted intelligent design and innovation. This development is moving towards system coordination and resource consolidation [11]. Technologies such as the Internet of Things, cloud computing, big data, and mobile Internet have enabled different functional structures in cities to coordinate with each other, resulting in intelligent, digital, and intensive urban management and operations. These advancements have provided people with more convenient services. Interactive landscapes embody the characteristics of intelligent cities, specifically the intelligent provision of services and interactive experiences. This facilitates collaborative and innovative development between the landscape and the intelligence of the city, as well as the provision of intelligent urban services [12]. The application of interactive technology in landscape design strengthens the connection between the landscape and people, space, and city. The use of digital media technology enhances the effect of the landscape and satisfies diverse needs of people.

The term “human–computer interaction” was first proposed in 1975, and its concept was popularized by the book The Psychology of Human-Computer Interaction by Card in 1983. The term “interaction design” was first proposed in 1984 by Bill Moggridge, one of the founders of IDEO [13]. Since then, scholars around the world have started to research and pay attention to this field. In 1997, Hiroshi Ishii and Brygg Ullmer from the MIT Media Lab proposed the concept of Tangible Bits, which involves coupling bits with everyday physical objects to allow users to directly grasp and manipulate them [14]. In 2013, Israeli scholars Oren Zuckerman and Avelet Gal-Oz concluded that due to the rich physical feedback information and more advanced forms of interaction brought by TUI (tangible user interfaces), it is recommended to promote the use of TUI-based interaction methods for human–computer information interaction [15]. Since then, many landscape architects, artists, and architects involved in human–computer interaction have been engaged in scientific research and design practices in this field, and research on interactive design of landscapes has followed.

Alan Cooper mentioned in his book The Road to Interactivity Design-Bring High-Tech Products Back to Humanity that combining scientific technology and digital media content with landscape design can provide people with a fresher experience [16]. In addition, Alan Cooper proposed in his book Software Concept Revolution: *The* Essence of Interaction Design that the core of interactive landscape design should focus on the relationship between people and the landscape [17]. His research emphasizes the leading role of people in landscape and interaction processes, which has become a core issue in later research.

Integrating the concept of “interaction design” into landscape design, using modern science, theory, and materials, and integrating new technological methods and artistic forms, can create landscapes that are in line with the characteristics of the site and meet usage needs. By involving people as components in landscape design, communication and interaction between people and the landscape can be achieved [18]. This facilitates the concentration of functions, the sharing of information, the integration of resources, and the participation of human intelligence. As part of urban construction, the research and development of interactive landscapes should constantly reshape the relationship between people and the world, provide new methods for addressing the shortcomings of traditional landscapes, and offer new pathways for fully integrating landscapes into urban systems. Applying the concept of intelligent design, interactive landscapes should continue to be developed with deeper research and broader applications.

### 2.3. The Necessity of Combining the Characteristics of MC Plant LD, CVT, and Multimodal Interactive Design

By analyzing the concepts of MU LD features, computer vision and multimodal interactive design, we find that there are similarities between these three.

Through the design of interactive landscapes in marine cities, landscapes and people can establish an organic connection, so as to effectively achieve the objectives of use and promote understanding and communication between people. To achieve this, however, it is important to follow the usability goals of interactive landscapes as well as the goals of good participant experience [19].

At this stage, marine cities are becoming more and more important in people’s daily lives, and the main purpose of MC plant LD is to provide tourists with places to visit and rest, so that the process of their experience can enrich their sensory experience and give them great satisfaction in both physiological and psychological aspects [20].

The two goals of usability of plant LD and good participant experience together serve the interactive LD of marine cities. The usability goal focuses more on the landscape product itself, while the participant experience goal considers the ideas and needs of people, and the two together promote the application and development of interactive LD in marine cities [21].

This paper selects computer vision software suitable for plant landscaping in ocean cities and proposes a general process for applying intelligent interactive technology to plant landscaping in ocean cities. This provides a certain supplement and improvement to the theoretical research in related fields and provides reference for the practice of multimodal interactive design of plant landscaping in ocean cities in the information age. Thus, it promotes the application of multimodal interactive technology in the landscape architecture industry. Practically, more research on the integration of the three has to be conducted:

(1) Beneficial for designers to refine their design proposals. Through the use of 3D and virtual reality (VR) interactive technology, designers can observe their design proposals in multidimensional virtual environments. This benefits them in conducting further analysis, deduction, and refinement of their design works, enabling them to promptly identify issues and reduce design flaws.
(2) Beneficial for presenting design proposals. Multimodal interactive technology has brought about a revolution in the field of landscape design representation. Viewers can not only intuitively experience the effects of the scene after construction, but also obtain more information from the scene through human–computer interaction, achieving information interaction. Moreover, virtual reality (VR) interactive technology supports demonstrations on both computers and mobile devices, greatly expanding the channels for presenting and communicating design proposals.
(3) Beneficial for communication between designers and decision makers. Through the use of computer VR multimodal interactive technology, property owners can view design proposals from multiple angles in virtual scenes according to their preferences, experience the site environment firsthand, and intuitively understand the designer’s design intentions. This is something that cannot be achieved with renderings, sand table models, or animations. After fully understanding the design intentions, property owners can communicate and negotiate more effectively with designers, and their opinions are also more targeted.

With the coming of the 5G era, intelligent design will further integrate and develop with global research and development, manufacturing, module, and other resources, enabling maximum information sharing and communication among all parts of the city. It will continue to evolve towards a more information-based, digitalized, and intelligent system. The most essential attributes of the landscape in ocean cities are interactivity and publicness. Therefore, the development of interactive landscapes plays an important role in the research and development of plant landscape design in ocean cities. Urban plant landscape design guided by interactive concepts always incorporates interactive thinking throughout the entire process, from conception to presentation. The rapid development of technologies such as big data, the Internet of Things, cloud computing, and artificial intelligence provides great possibilities for the realization of smart landscapes, promoting the development of the “smart” concept. Future explorations of smart spaces based on sustainable development principles will become an important trend in the development of ocean urban landscapes [22].

### 2.4. MU LD Based on Multimodal Interaction

(1) Interactive water body LD

The water body LD of marine cities takes interactive design as the core, considers the relationship between water body landscape and terrain, people, and roads, and coordinates the relationship between them to achieve good communication and an interactive experience between people and the water body landscape.

Relationship between the water landscape and the site: When designing the water landscape of the ocean city, the topography, soil, climate, and water table of the ocean city will be surveyed and investigated in the field to determine whether the location is suitable for developing the waterscape and how to design the waterscape. Secondly, we should focus on how the water feature is related to human recreational activities, and combine human behavior and psychological needs to design a reasonable interactive water landscape.

Relationship between water landscape and roads: There is also a close relationship between water landscape and roads in urban parks. Roads are the main way for people to enjoy waterscapes, as they connect all waterscapes in the city [23]. Based on the concept of interactive design, people are guided to carry out hydrophilic activities through landscape facilities such as plank roads and Tingbu, so that visitors can have a relaxing and pleasant experience in the ocean city.

**Figure 2:**
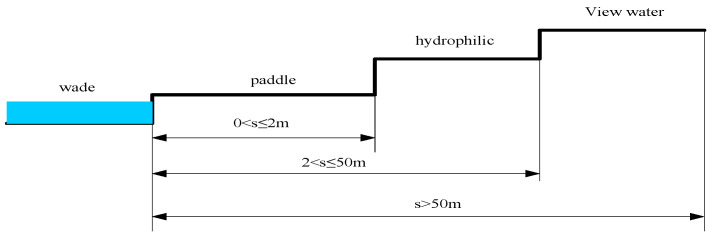
The effect of distance on human hydrophilicity.

Relationship between water landscape and people: It is human nature to be hydrophilic and to be close to water. Therefore, landscape planners should increase opportunities for interaction between people and the water landscape, such as wading, playing in water, being hydrophilic, and watching water. As shown in Figure 2, when people enter the water to experience it, this is considered wading activity. When the distance between people and water is 0 < s ≤ 2 m, it is playing in water activity. When the distance between people and water is 2 < s ≤ 50 m, people are considered to be hydrophiliced, and if the distance between people and water is >50 m, people are engaging in water-viewing activity. These activities can make people have a variety of waterscape experiences and can narrow the distance between people and the waterscape.

(2) Interactive landscape facilities design

The concept of interactive design is introduced into the design of landscape facilities, adhering to the principle of interactive participation, providing quantitative services for someone visiting the marine landscape, and promoting a good interaction between people and facilities [24].

First, the design of interactive landscape facilities should be human-centered. In the design, designers should always consider the functional use by people, so that people’s needs are satisfied through the landscape facilities. For example, guiding signs and advertising boards of the ocean city guide visitors on tour routes and provide science education, and ocean sunscreen chairs provide opportunities to rest.

Second, the design of interactive landscape facilities should be universal. Generic designs should mainly consider disadvantaged groups, e.g., the mobility and hearing impaired, pregnant women, or individuals with chronic diseases, among other groups of people, and they should be concerned about taking care of their psychological emotions and increasing their use of landscape facilities.

Next, the design of interactive landscape facilities should be easy to use. First, the spatial scale should both meet the psychological and usage needs of people and be scientific and reasonable. For example, seats or platforms for resting should be set up along longer roads and at corners in marine cities to buffer the flow of people. Second, human scale refers to the corresponding design for people of different ages; for example, there is a great difference in height and ability between children and adults; therefore, the design cannot consider only adults and ignore the use of landscape facilities by children.

(3) Interactive plant LD

The interactive design is introduced into the MC plant LD to meet the basic psychological needs of people at the core, to create a landscape space with a sense of hierarchy and richness for visitors, and to increase the chances of visitors interacting with marine plants and animals or other elements [25].

Plant LD should be pleasant. In the selection of plants, plants of different sizes are planted according to the size of the activity site. For example, at a site where visitors are concentrated, large-sized trees should be planted, which can provide shade and shelter for visitors and a good interactive environment.

## 3. Case Design of Coastal Plants in Sanya Bay, China

### 3.1. Assessment and Analysis of Marine Eco-Economic Zoning in Sanya Region

#### 3.1.1. Analysis of Spatial Heterogeneity

As shown in Table 1 below. Considering the sub-indexes of the three subsystems in the Sanya marine eco-economic system—namely, marine ecology, marine economy, and marine society—an evaluation index system for marine eco-economic zoning was built and the marine ecological economy of Sanya was evaluated via zoning using comprehensive factor evaluation and analysis. Briefly, this process was divided into three steps. First, a zoning evaluation index system was built. Second, the weight of each index was calculated and determined by issuing questionnaires and scoring by experts. Third, rational values were assigned to concrete indexes for each region according to the evaluation criteria. Finally, the evaluation index was obtained via calculations and the zoning evaluation was made.

#### 3.1.2. Data Source

**Figure 3:**
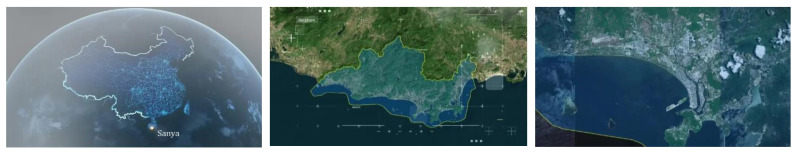
Map of Sanya.

The research data used in this study primarily included Google satellite images, previous urban master plans for Sanya, other specialized plans, and social and economic development plans. Amid studies on the urban form of Sanya City, our work mainly referred to the compilation year of the latest version of Sanya’s urban master plan, and we processed data using three main reference pictures that depended on the data collection time of the Google satellite map; that is, the status quo image from 2014 (Figure 3).

### 3.2. Case Study of Coastal Plants in Sanya Bay—Anaya Community

#### 3.2.1. Design Ideas

**Figure 4:**
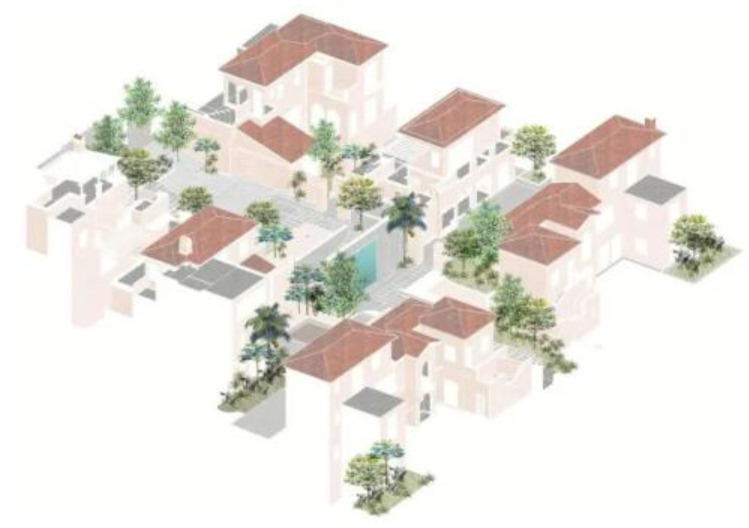
Landscape distribution of Sanya Anaya community.

Major goals of design ideas are to adhere to the people-oriented concept, carry out interactive landscape design of the ocean city, pay attention to how the landscape is integrated into people’s life and environment, have landscape usability and good user experience as the goal, and promote good communication and interaction among people and between people and the environment. Based on the design goal of a multi-modal interactive landscape of the ocean city, this design project carries out reasonable planning and layout for the functional zoning, road system, water landscape, landscape facilities, and plant configuration of the Sanya Bay Anaya community, providing people with different functional requirements and bringing different viewing experiences, as shown in Figure 4.

#### 3.2.2. Functional Zoning Design Based on Tourists’ Needs

The word “Aranya” comes from the Sanskrit word “Aranjo”, which means “a quiet, idle, remote, or secluded place for spiritual practice away from the hustle and bustle of human activity” [26]. Aranya has also come to Sanya, advocating for an aestheticism that believes “life can be more beautiful,” and arousing a great deal of curiosity among the people living in the mountains and seas of Sanya about the aesthetics of life. Combined with the development of the MC and the needs of tourists, based on the distribution of marine natural resources, the MC is divided into four functional areas: the entrance landscape area, the ornamental tour area, the water-friendly landscape area, and the quiet rest area.

(1) Entrance landscape area

**Figure 5:**
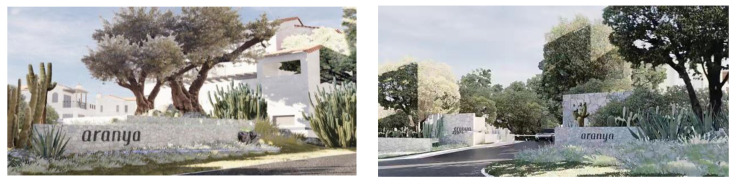
Sanya Anaya Entrance.

As shown in Figure 5, The entrance landscape area is also the service management area of the Sanya Anaya scenic spot. It is located in the axis of the scenic spot and connects all functional zones, so that visitors can enter the scenic spot as soon as possible to carry out their favorite activities in the area. This area also includes ecological parking lots, visitor service centers, and other landscape facilities to meet the physical and psychological needs of visitors. The design of the entrance is such that by minimizing the entrance of the scenic area, when visitors enter the scenic area, they are met with a large and open space. This contrast reflects the image of “small entrance, large landscape”, giving visitors a visual impact, with rich visual level changes to guide visitors to interact with the landscape. Electronic guidance devices are also set up at the entrance, and VR panoramic photography is carried out for the whole scenic area, so that visitors can scan it on their cell phones and experience all the scenery of the scenic area, making it convenient for them to choose the functional area they want to go to.

(2) Viewing tour area

**Figure 6:**
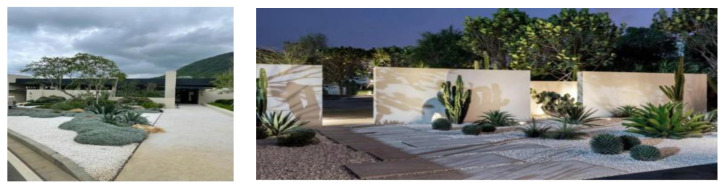
Anaya Scenic Area.

As shown in Figure 6, the Aranya in Sanya is full of tropical plants with a distinctive Middle Eastern ‘flavor’. The architecturally designed buildings are reminiscent of Tuscan-style estates, and the garden is filled with exotic cacti. Visitors entering the Ocean City Scenic Area can immerse themselves in the ocean culture and the wonderful ocean scenery, creating an immersive experience for visitors with multi-sensory means. By utilizing plant landscape design, visitors’ attention can be focused on interacting with the scenery, achieving mutual communication between people and plants. Different plants are in varying growth states, resulting in different visual effects and leading to multimodal visual expression.

(3) Water-friendly landscape area

**Figure 7:**
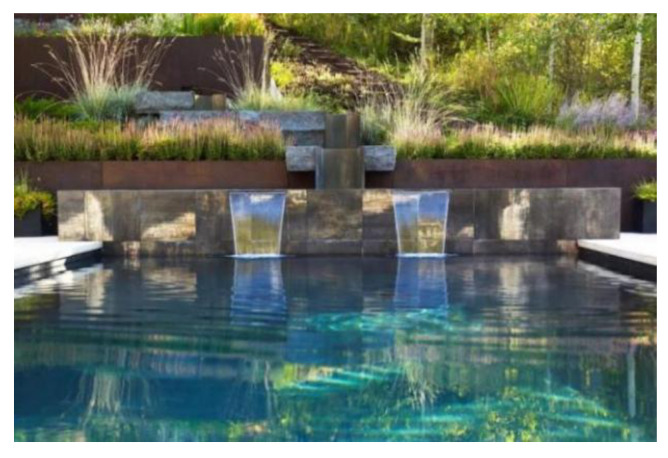
Anaya Water Plank Path.

As shown in Figure 7, according to the topographic characteristics of the ocean city, water-friendly landscape areas are set up in areas with large water surfaces, advocating the “close to the ocean nature” way of touring to meet the needs of tourists. Visitors can reach various spots through water stacks and circular walkways, which have good hydrophilicity and experience, and guide visitors to interact with the water body landscape tactilely. Specific strategies include, firstly, improving accessibility and enhancing the transportation connection between coastal plants and water. The second strategy is to enhance visual accessibility and minimize the obstruction to the water views by plants, as unobstructed views are another important means of increasing the water-friendly environment. The third strategy is to bring water ashore and introduce water into the interior of the waterfront space for landscape shaping, achieving a coordinated and harmonious effect between the interior and exterior spaces and greatly enhancing the waterfront space’s affinity with water. The fourth strategy is to enhance the psychological experience of being near the water. When designing the plant layout, elements related to water, such as aquatic creatures, wave shapes, boat shapes, and color elements, can be used in the surrounding landscape facilities to enhance the psychological experience of visitors being near the water [26].

(4) Quiet rest area

**Figure 8:**
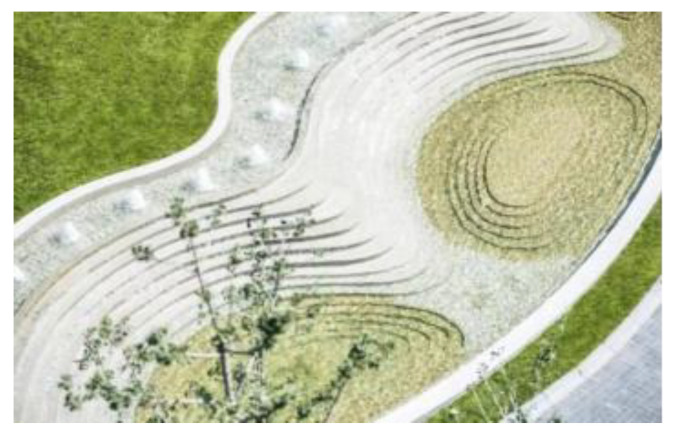
Anaya vegetation area.

As shown in Figure 8, The quiet rest area is designed especially for visitors, to provide people with quiet rest and interaction activities. This area includes the marine hotel area and a camping area. The marine hotel provides a resting place for visitors, and the beautiful scenery around the hotel stimulates visitors’ vision and offers a good viewing experience. The camping area is on the open beach, providing visitors with space for casual activities. They can freely contact the sea breeze, waves, and the surrounding environment, and feel the good experience of contact between humans and nature, thus creating a behavioral interaction.

#### 3.2.3. Five-View Plant System

**Figure 9:**
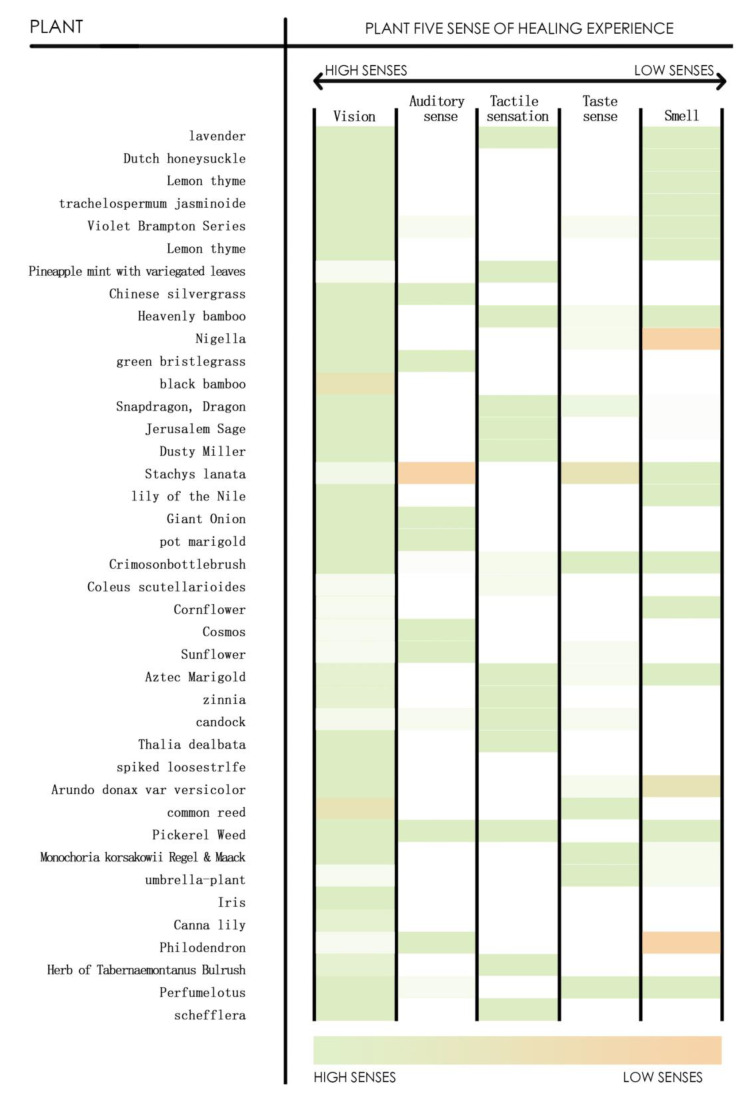
Statistical Diagram of Plant Five Sensory Modes.

When using plants to create an ocean resort landscape, it is essential to consider the physical and psychological sensitivity of the experience [27]. By selecting and using different plants through scientifically rigorous design, human experiences can be incorporated to create a plant landscape that excels in the five senses of “sight, sound, smell, taste and touch”. Only then can a maritime landscape space be created that combines aesthetic taste with leisure and entertainment functions, as shown in Figure 9, which presents a statistical summary of the five sensory modes of plants.

- Visual effect

Plants with warm tones can create a sense of warmth and evoke positive emotions, while plants with cool tones can help calm down individuals who are feeling restless or anxious. When designing plant landscapes, a diverse selection of species should be considered to meet various landscape preferences. As for plant variety, the crown, color, season, and form of cultivation should be comprehensively considered to create a rich visual effect.

- Auditory effect

In 1929, Finnish geographer Grano first proposed the concept of “Soundscape” to research the audience-centered sound environment [28]. Different sounds bring completely different feelings, so a design should try to isolate noise and make more use of natural sounds to create a favorable auditory landscape. Natural sounds such as pine-soughing valleys, fine rain in a bamboo forest, windy lotus in a winding courtyard, and rain drops drumming rhythmically against banana leaves create classic auditory landscapes. A special area can be designated as a meditation space in the ocean vacation area to plant a bamboo forest. Visitors can relax and alleviate their anxiety while experiencing the rustling sound of bamboo leaves swaying in the wind within the bamboo groves.

- Smell effect

Using the fragrance emitted by plants or creating an atmosphere with their scent can create a healthy and clean landscape environment with a therapeutic effect. There are two types of plants: sterilizing, air purifying plants and external treatment functional plants.

- Gustatory effect

The taste effect is usually achieved by combining the landscape environment with experiential activities. Small tourist orchards and agricultural parks can be built, where various fruits and vegetables can attract the elderly to leisurely taste and enjoy the produce, thereby adding a taste experience and relaxing their mood.

- Tactile effect

Among the five senses of sound, sight, smell, taste, and touch, touch is the most difficult to grasp. It requires people to experience an area through touch in order to stimulate their physiological responses and convey emotions. This effect often needs to be achieved through plants with good texture.

**Figure 10:**
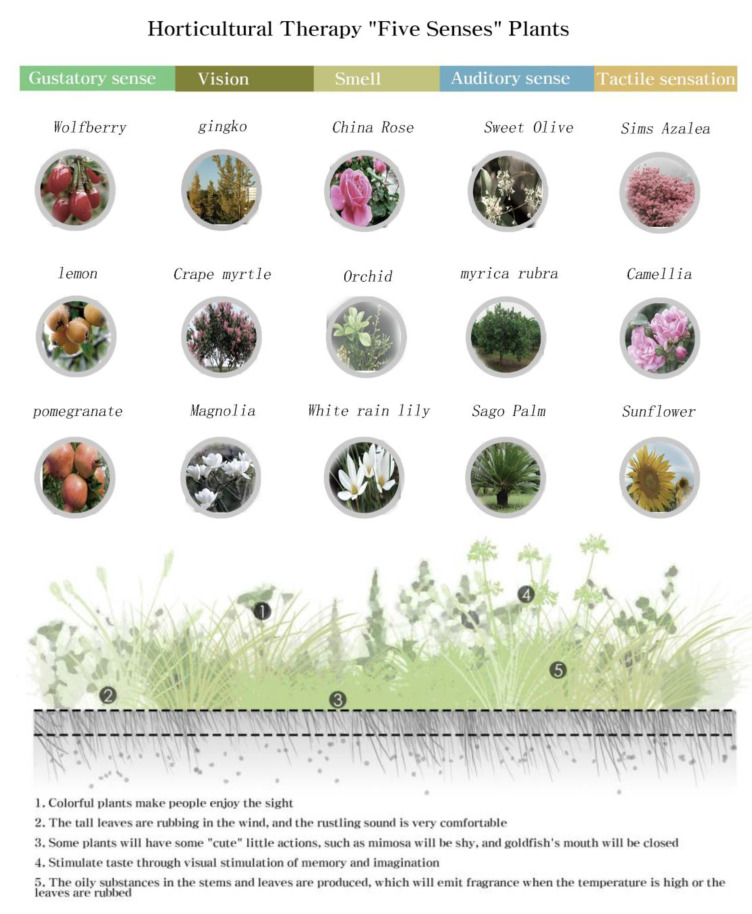
Multimodal configuration of plants.

In the plant landscaping of marine cities, the modal configuration of different sensory systems produced by plants is summarized as follows, as shown in Figure 10.

## 4. Evaluation of the Interactional Impact between CVT, MC Planting, and MID

### 4.1. Respondents’ Occupational Distribution

As shown in Table 2 below.The respondent population was divided into three parts: working professionals, students studying landscape architecture, and non-landscape architecture professionals. As far as possible, a certain sample size was ensured for different groups of people to better analyze and compare the views of different respondents and to ensure more objective and accurate results.

### 4.2. Evaluation Analysis of the Effect of Computer Vision-Based MID

When evaluating the effectiveness of the design approach in this paper, the evaluation degree was divided into “strongly disagree”, “disagree”, “not sure”, “agree“, and “strongly agree”, with scores of 1, 2, 3, 4, and 5, respectively. This method was used to quantify and analyze the respondents’ answers.

**Figure 11:**
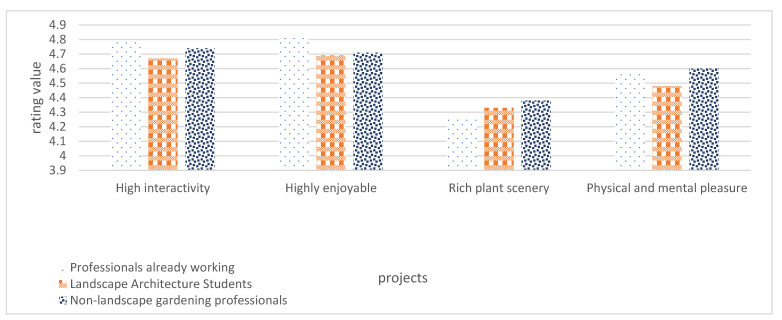
Evaluation of design effects.

As shown in Table 3 and Figure 11, the respondents’ ratings for each item exceeded 4.00, indicating that the respondents were satisfied with the effectiveness of computer vision-based MID. Among them, the respondents agreed more on “Computer vision-based MID is interactive” and “Computer vision-based MID is ornamental”, with mean scores of 4.73 and 4.74, respectively. The lowest score is “rich content of plant scenery”, with an average score of 4.32. There are two main reasons for this: first, the current common modeling software has a limited ability to restore the scene, and the realistic feeling of plant models is not strong, which is the main reason for the low score; second, the accuracy of the case scene modeling still needs to be improved.

Among the three groups of people interviewed, the scores given by landscape architecture students were generally low, probably because of the large number of students interviewed and the large difference in their evaluation of the case presentation effect, which was reflected in the fact that more respondents chose low scores, thus leading to a decrease in the average score.

### 4.3. Attitudes toward the Expression of Computer Vision-Based Multimodal Interactive Design

**Figure 12:**
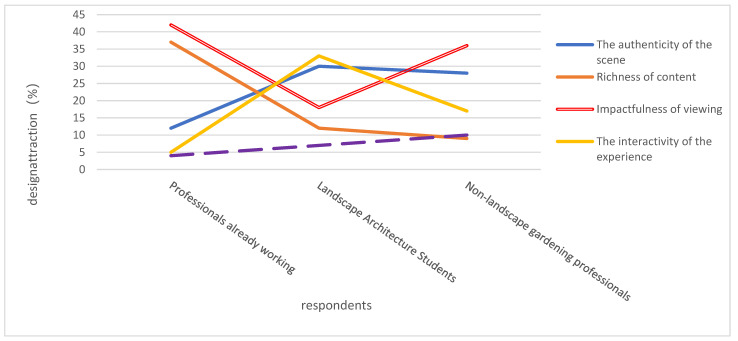
Design attraction points.

As shown in Table 4 and Figure 12, the most attractive features of computer vision-based multimodal interactive design for plantscapes are the richness of content (37%) and the impact of viewing (42%), probably because working professionals pay more attention to the role of CVT as an aid to their plant LD work, and it just so happens that the richness of information and novelty of computer vision expressions are very suitable for them. Therefore, they choose “richness of content” and “impactfulness of viewing” the most.

For landscape architecture students, their concerns were more dispersed, with slightly more people choosing “realism of the scene” and “interactivity of the experience”, at 30% and 33%, respectively. Among the three categories, landscape architecture students preferred “interactivity of experience” much more than the other two categories, probably because the interactive points in the display case contain plant information, which is helpful for students to understand and learn plant characteristics, so the interactivity in the multimodal design display is more attractive to them.

For non-landscape professionals, “impact of viewing” (36%) and “realism of the scene” (28%) were the most attractive features of computer vision and multimodal interactive design displays, i.e., 64% of non-landscape professionals were attracted to computer vision displays. The reason for this is probably because non-professionals lack the appropriate professional knowledge and rely more on visual images to understand the designer’s intention, and are more interested in the outcome of the design than the rationality of the design, so they are more impressed by the visual experience brought to them by computer vision displays.

### 4.4. Interactivity throughout the Design Process

**Figure 13:**
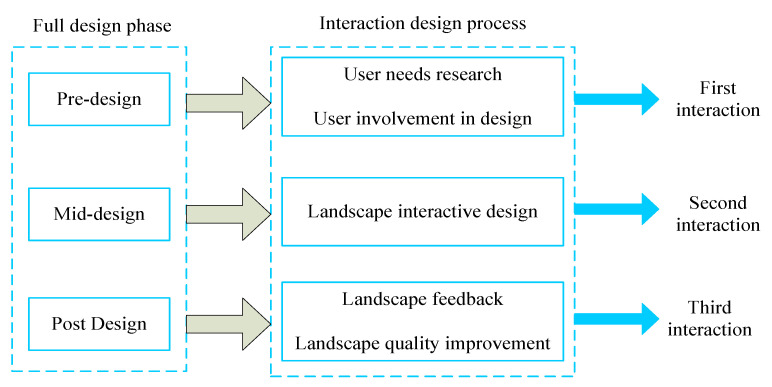
Three processes involved in interaction design.

The multimodal interactive design of MU planting based on CVT can be divided into three processes: pre-design, design process, and post-design [29]. As shown in Figure 13, in the pre-design stage, extensive and detailed information collection is required, including landscape general style cases, user research and analysis, and information collection on the current situation of the plot. This process involves the first interactive design, which strengthens the communication with tourists in the research process, and derives the general or special needs of tourists from the psychological analysis of the researchers, indirectly making tourists participate in the LD. In the middle of the design, with full consideration of user experience and interaction, a detailed design of various parts of the landscape such as plants, paving, landscape facilities, etc., increases the direct interaction between visitors and the landscape, which is the second interaction process between landscape and people. In the late design stage, in the property customer service department, through the visitors’ information feedback on the landscape, the landscape will be modified for quality improvement, which is the third interaction process. Therefore, the mutuality design of the landscape in the study area should follow this principle, so that interactivity occurs throughout the design process.

### 4.5. Data Result Analysis

(1) Using willingness analysis

**Figure 14:**
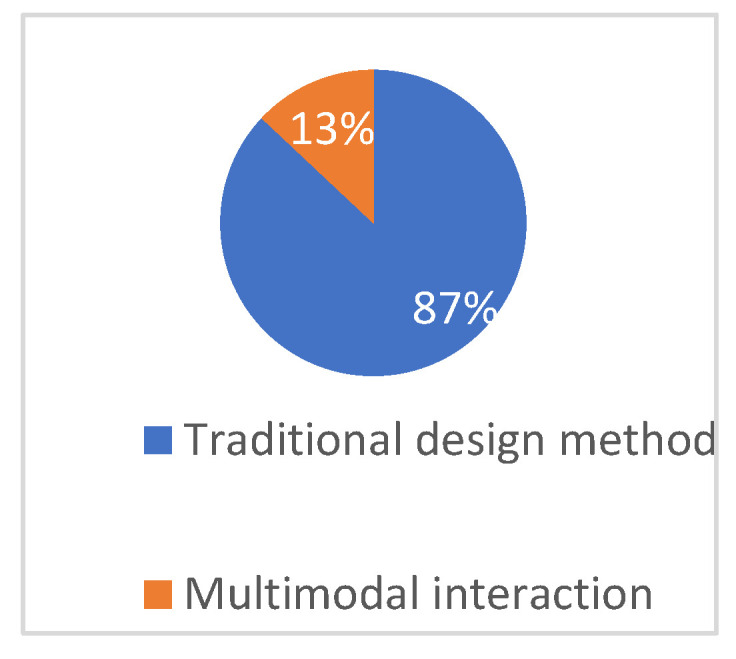
Analysis of preference for design means.

**Figure 15:**
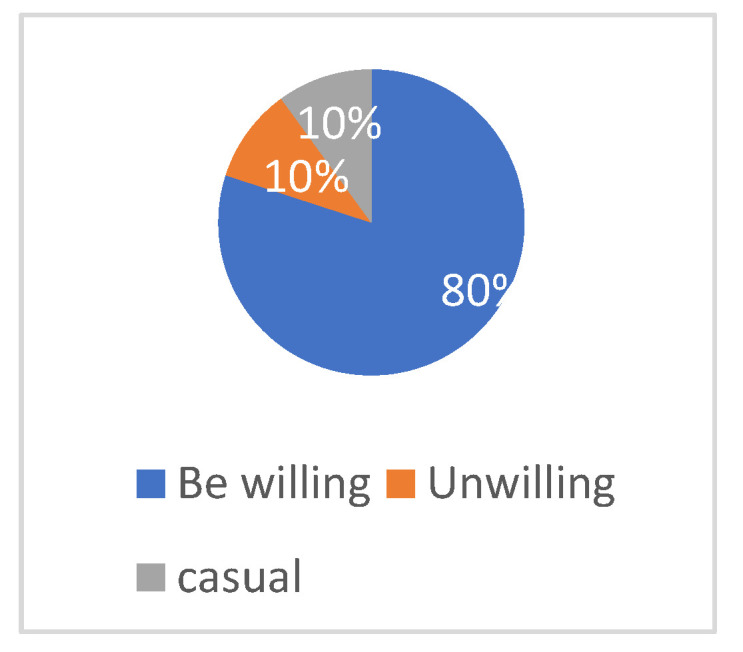
Analysis of willingness to use interactive technology.

According to Figure 14, although people working or studying in the field of landscape architecture have a general understanding of multimodal interaction technology, most of them (87%) still prefer to use interactive technology for design when possible. This also indirectly indicates the professionals’ liking and affirmation of VR multimodal interaction expression methods. As shown in Figure 15, for non-professionals who are not familiar with landscape design, most people (80%) are still willing to try interactive technology to experience the plant landscape space in the ocean city.

(2) Expectations for the future application of VR multimodal interaction technology

**Figure 16:**
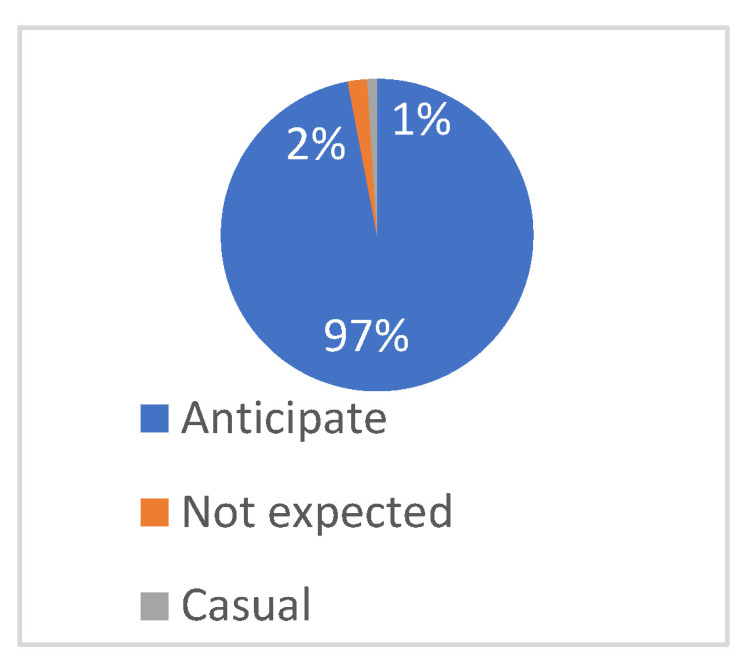
Interviewees’ expectations for the application of multimodal interactive technology in plant landscape in the future.

According to Figure 16, as many as 97% of the respondents expressed their expectations for the future combination of multimodal interaction technology and the field of landscape architecture, indicating that interactive technology is highly popular among different groups of people.

(3) Statistical Differences in Respondents’ Willingness

The basic formula of the χ^2^ test, according to the respondents, is χ^2^ = ∑(1 − α)^2^/α; the χ^2^ value reflects the degree of agreement between the actual frequency and the theoretical frequency. If H0 holds, the difference between 1 and α should not be huge; the χ^2^ statistic should not be extensive. The more significant the difference between 1 and α, the larger the χ^2^ value and the smaller the corresponding *p* value. According to the *p* value obtained by the significance test method, generally, *p* < 0.05 is considered to have a statistical difference, *p* < 0.01 is deemed to have a significant statistical difference, and *p* < 0.001 is regarded as a highly significant statistical difference. These values mean that the probability of the difference between samples being caused by sampling error is less than 0.05, 0.01, and 0.001, respectively [30]. According to the specific analysis of the chart, it is concluded that, as shown in Table 5, there is the most significant statistical difference in the statistics of non-landscape professional groups. The reasons are that the groups have different occupations and considerable age differences, and are influenced by their living environment. Secondly, students majoring in landscape architecture have apparent differences in applying technical means due to other individual concepts. Finally, there are working groups in this profession to develop more and better design methods, and there is a slight difference in willingness.

## 5. Discussion

### 5.1. Comparison of Relevant Literature Results

A Literature Research Results

There are few studies abroad on the multi-modal interaction and landscaping of garden plants, and most are biased towards the protection, utilization, and ecological research of wild plants. The book *Landscape Plant Configuration* by British scholar Brian Clouston discussed the design of trees, shrubs, ground cover plants, herbaceous plants, aquatic plants, and forest planting from the perspective of ecology and aesthetics [31]. The maintenance and management of gardens combines garden design, construction, and maintenance management, and puts forward the viewpoint of a four-dimensional management scale, systematically organized in the book *About Face: The Essentials of Interaction Design* by American scholar Alan Cooper [32]. Regarding some design concepts of digital product interaction design, a more detailed introduction to the application of computer vision technology, and elaboration on plant landscape design, plant planting and maintenance, especially the close relationship between landscape and environment, is provided [33]. The scientific and artistic importance of design has been analyzed in detail; the discussion on the functions of plant materials and planting design in the book Elements of Landscape Architecture Design written by American scholar Norman K. Booth in 1983 has become the basis of plant landscape construction, especially in the classic basic theory of plant space construction [34].

The research and attention on multi-modal interaction design technology in landscape architecture are gradually increasing. Zeng Junfeng and Qiu Cuiju discussed the application of interactive technology in garden design earlier in the *Chinese Garden* magazine. Li Guosong et al. used the Analytic Hierarchy Process (AHP) to evaluate the interactive realization method of plant construction under the five sensory modes [35]. Thus, the sense of touch is the best way to realize the interactive form of landscape garden plants. In terms of theoretical research, there are mainly the following essential papers: “Interactive Design and Visualization of 3D Plant Models”, “Interactive Plant Modeling System”, and “Forward Interaction: From Voice, Gesture Design to Multi-modal Fusion”, “Chinese Garden Plant Landscape Art” by Professor Zhu Junzhen, and “Garden Plant Landscape Design and Construction” edited by Zhao Shiwei, etc. all have high academic value [36]. These studies systematically summarize the development process, design principles, and theoretical basis of multi-modal interaction, guiding people to explore new interactive experiences. They also analyze the characteristics and limitations of each modality and outline the design model of multi-modal interaction, from voice and gesture design to multi-mode fusion, building a graphical voice interface fusion model with growth capabilities. Although corresponding progress has been made in theory, many problems remain. In theoretical research, there is no systematic theory to guide practice: the practice is too limited and stays in the local environment, and the city as a whole is not considered uniformly.

B Research results of this article

This paper integrates design cases with interactive technologies, highlighting the immersive, interactive, and imaginative advantages of multimodal interaction design in plant landscaping through the exchange of people’s “five senses”(Table 6). This is significantly different from the traditional focus on plant adaptability and environmental protection in landscape planting.

(1) Immersive scenario experience. Compared with traditional plant landscape creation, computer vision technology immerses people directly in a multimodal three-dimensional space, achieving the effect of being on the scene in person [37]. The immersive scenario provides a new visual and sensory experience for both professionals and the general public, adding to the dimension of the designer’s exploration space. The information received by the brain in daily life is caused by multiple senses, so it is impossible for people to have an immersive experience if it is just visual immersion. With the aid of necessary computer vision and interactive devices, it is possible to achieve sensory immersion beyond visual cues by incorporating auditory and olfactory cues, allowing visitors to experience the ambiance of different botanical landscapes. It adds a weather conversion function to the system to simulate natural landscapes such as rain, snow, and fog. In cooperation with spatial audio technology, the unique plant landscapes of the ocean city can be felt even while sitting at home.
(2) Unobstructed view space. In addition to the immersive visual experience, computer visual interaction technology also provides people with the opportunity to view space freely. Renowned British urban planner Gordon Cullen, in his book *Cityscape*, mentions that understanding space is not only about looking at it but also about moving through it [38]. With the help of computer vision and interactive technology, users can simulate a coastal visit, observing the form and texture of plants up close. In the past, designers typically used hand-drawn plans, perspectives, and even physical models to aid in the exploration of plant landscape spatial structure design. However, the creation of landscape spatial sequences stems from the continuity of human behavior, which reflects the “human-centered” design concept.
(3) Real-time feedback on modifications. The lack of computer precise positioning design will lead to a lack of judgment on the on-the-spot environment, which leads to a lack of aesthetics in plant landscaping, and even the inability of plant interspersed design to interact with people [39]. Aided by computer vision, the plant scene is designed to see effects at any time scale. This ensures that landscape designers can make timely modifications and compare effects when design errors are discovered, ultimately resulting in the most suitable plan. Since plants are different from buildings that possess a vital nature, plants of the same species with different sizes can have an important impact on the landscape space and the effectiveness of the landscape [40]. The advantage of setting and modifying scene elements in real time is that the inspiration experienced by the designer during interactive editing is saved to ensure the continuity of design thinking.

### 5.2. Research Innovation and Significance

The case design analysis above validates the credibility of previous research and the results of multimodal interaction [41]. The use of multimodal interaction technology will enable close integration of the relationship between plants in ocean cities and tourists, resulting in that when people walk into the landscape, plants are not only the landscape through which people travel; rather, they experience a new type of landscape. The multimodal interaction study of computer vision technology in plant landscaping in ocean cities is not only of practical significance for the design of plant landscapes, but also has multi-aspect research significance for future interaction research fields [42]. After using computer visual interaction technology, plant landscape design reinforces the communication and interaction among people, and between people and scenery, and its innovative value is mainly reflected in three aspects: the expansion of design expression tools and display methods, the gradual innovation of traditional thinking, and the realization of multiple landscaping effects.

(1) Expand the design performance tools and display means. The visual interactive technology craze has broken the shackles of two-dimensional expression, placing the design process in a multi-dimensional computer scenario, with the application of new technology signifying the intervention of new tools [43].
(2) Traditional ways of thinking are gradually reforming. Existing examples show that the emergence of new software can not only break through the limitations of past design expressions, but can also change design thinking, such as the introduction of parametric software that has increased landscape plant design from perceptual art creation to a combination of perceptual knowledge and logical thinking [44].
(3) Diversified landscape effects. The five senses of plant characteristics are brought into the plant landscape design, which adds more design principles and sensory experience to the original single-plant layout to achieve conservation and aesthetic improvement of the plant environment in the ocean city.

### 5.3. Research Limitations and Future Research Routes

This case study attempts to integrate people’s multi-sensory experience with plant design through the introduction of computer vision technology, achieving a visually modernized plant landscaping performance. The creative plant configuration method tailored to different visitor experiences was explored through a questionnaire survey. However, there are still some limitations to the research that need to be further strengthened in the future.

(1) There is currently insufficient in-depth research on the literature of computer visual interaction technology related to plant landscaping, which is still a field that scholars should continue to explore more deeply.
(2) Plant landscapes in different ocean cities may be influenced by climate and geographical location according to different regional characteristics, which will lead to differences in plant matching designs according to different ocean cities.
(3) Since multimodal interactive plant design is mostly determined by people’s sensory consciousness, this does not represent the most appropriate design method for the whole case, and there are inevitable limitations based on the analysis of the results of different people’s design experiences.

In light of the limitations of the above research, the effect presented by multi-modal interactive technology is not enough to satisfy people. There is still a distinct difference from the ideal state, which objectively hinders the combination with its landscape environment [45]. The emergence of tools is necessary to meet people’s needs. If the interactive technology software cannot provide enough support for garden plant landscaping, it may be as short-lived as the once-popular 3D TV and eventually disappear in the landscape industry. Therefore, in the future, designers will play a more significant role in promoting than programmers in the development process of the combination of the two. Designers should explore and consider the application potential of multi-modal interaction and provide more constructive suggestions for software development with their professional sensitivity [46]. In the future, the VR virtual interaction software used in garden plant landscaping should integrate scene modeling, design deliberation, and post-production performance. The model does not need to be converted between various software. The software should be intuitive and easy to operate, allowing designers to focus on the design and express their thoughts and ideas through VR virtual interactive software without hindrance [47].

## 6. Conclusions

A perfect plant landscape needs to meet the unified combination of scientific and artistic principles, i.e., it should meet the survival habits of plants in the regional environment and the embodiment of the plants in terms of morphological appearance [48]. It is necessary to start from the principle of artistic matching, to show the visual effect of plants alone or combined with other plants, and most importantly, to make people appreciate a wonderful sense of immersion. This paper introduces CVT and MID theory into the design of MU planting, adding a new design tool to the construction of marine cities. The use of computer vision technology brings three major characteristics to plant landscape design: immersion, interactivity, and creativity, providing a multi-sensory, multi-environment, and cross-temporal immersive experience based on the original two-dimensional plant configuration design. Computer vision interaction technology has not only become a new way to showcase urban plant landscape display solutions, but also a tool to assist designers in thinking and inspiring new design ideas or concepts, ultimately improving the quality of the design. By analyzing and discussing the various types of MU botanical landscapes, it shows the artistic effect of CVT and MID in botanical landscapes. The study shows that as a design worker, one should know how to analyze and solve the problems in the landscape from the audience’s point of view, and be able to meet people’s needs for ornamental, interactive, and botanical landscape content enrichment and physical and mental pleasure of MU botanical landscapes, so as to better realize the communication between humans and nature [49]. It is hoped that the research in this paper can add some constructive ideas and strategies to the construction of marine cities in the future. Through this process, people can live in a more perfect urban space, so that the art or technology of plant landscaping in the whole MC can be gradually enhanced and continued.

## Figures and Tables

**Table 1:** Evaluation index system of marine ecological suitability.

Target Layer	Criterion Layer	First-Level Indicators	Secondary Indicators	Element Layer	Direction	Required/ Not Required
Marine ecological suitability	Suitability of marine ecological environment	Biological environment structure	Environmental quality	Eutrophication index	Negative indicator	●
			Natural shoreline integrity	Natural shoreline retention rate	Positive indicators	●
			Marine disaster risk distribution	Marine risk intensity index	Negative indicator	○
			Community structure	Biodiversity index	Positive indicators	●
		Biological environment function	Important protection value	Ecological sensitive areas and important protected objects	Positive indicators	●
			Landscape type	Ecological disturbance degree	Negative indicator	●
			Production supply	Net primary productivity	Positive indicators	●
	Suitability of marine social environment	Current situation of space utilization	Deepwater shoreline resources	Available deep-water shoreline length	Negative indicator	●
			Mudflat resources	Available mudflat area	Negative indicator	●
			Landscape cultural resources	Rating coefficient of scenic spots	Negative indicator	○
	Suitability of marine economic environment	Socio-economic conditions	Marine economy	Total output value of marine industry	Negative indicator	●
			Coastal population	Population density	Negative indicator	●
			Geographic conditions	Accessibility of important nodes	Negative indicator	●

● means that this option is a mandatory choice for the Evaluation index system of marine ecological suitability. ○ means that this option is a non-mandatory option of the Evaluation index system of marine ecological suitability.

**Table 2:** Distribution of Respondents.

	Professionals Already Working	Landscape Architecture Students	Non-Landscape Gardening Professionals
Number of people	20	50	30
Proportion	18%	55%	27%

**Table 3:** Respondents’ evaluation of the effectiveness of computer vision-based MID.

	Professionals Already Working	Landscape Architecture Students	Non-Landscape Gardening Professionals
High interactivity	4.78	4.67	4.74
Highly enjoyable	4.82	4.69	4.71
Rich plant scenery	4.26	4.33	4.38
Physical and mental pleasure	4.57	4.48	4.60

**Table 4:** Analysis of attraction points of MID expressions based on computer vision (%).

	Professionals Already Working	Landscape Architecture Students	Non-Landscape Gardening Professionals
Authenticity of the scene	12	30	28
Richness of content	37	12	9
Impactfulness of viewing	42	18	36
Interactivity of the experience	5	33	17
Nothing particularly impressive	4	7	10

**Table 5:** Statistical differences in respondents’ willingness.

Target Statistics	*p* Value	Professionals Already Working	Landscape Architecture Students	Non-Landscape Gardening Professionals
Acceptance of interaction concept	*p* ≤ 0.001			●
	*p* ≤ 0.01	●		
	*p* ≤ 0.05		●	
Diagram schematic analysis		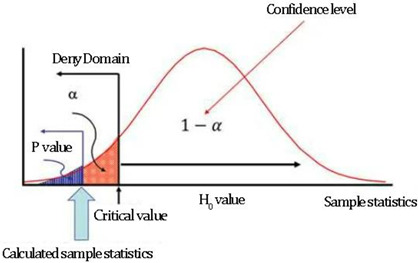		

**Table 6:** The embodiment of multimodal interaction characteristics in plant landscape design of marine cities.

Characteristic	Basic Content	Embodiment in Plant Landscaping of Marine Cities Based on Multimodal Interaction
Immersive	Multi-dimensional perception: observe the scene personally	Assisting in scheme deliberation and feeling plant landscape artistic conception: increasing the dimension of space exploration between designers and the public
Interactivity	Manipulate virtual reality scene: interact with the scene	Changes and contrast schemes that are conducive to designers’ feelings: save and verify the fleeting design inspiration in time.
Conceptual	Deepen concepts or sprout new ideas in the observation and manipulation of objects	Inspire the production of new design ideas or concepts

## Data Availability

The raw data supporting the conclusions of this article will be made available by the authors, without undue reservation.

## References

[1] Schiavo G., Mich O., Ferron M., Mana N. (2022). Trade-offs in the design of multimodal interaction for older adults. Behav. Inf. Technol..

[2] Weitz K., Schiller D., Schlagowski R., Huber T., André E. (2021). “Let me explain!”: Exploring the potential of virtual agents in explainable Al interaction design. J. Multimodal User Interfaces.

[3] Harrington M.C., Bledsoe Z., Jones C., Miller J., Pring T. (2021). Designing a virtual arboretum as an immersive, multimodal, interactive, data visualization virtual field trip. Multimodal Technol. Interact..

[4] Pietrzak T., Wanderley M.M. (2020). Haptic and audio interaction design. J. Multimodal User Interfaces.

[5] Kumar N., Belhumeur P.N., Biswas A., Jacobs D.W., Kress W.J., Lopez I.C., Soares J.V. (2012). Leafsnap: A computer vision system for automatic plant species identification. Proceedings of the Computer Vision–ECCV 2012: 12th European Conference on Computer Vision.

[6] Li L. (2020). Features and discourse analysis of English for plant protection under multimodal theory—A review of English for Plant Protection. J. Plant Sci..

[7] He L., Rie S. (2022). The Integration of Visual Modeling and Interactive Technology of Virtual Landscape Architecture. Proceedings of the Application of Intelligent Systems in Multi-Modal Information Analytics: The 4th International Conference on Multi-Modal Information Analytics (ICMMIA 2022).

[8] Angelini L., Caon M., Caparrotta S., Khaled O.A., Mugellini E. Multi-sensory EmotiPlant: Multimodal interaction with augmented plants. Proceedings of the 2016 ACM International Joint Conference on Pervasive and Ubiquitous Computing.

[9] Li Z., Guo R., Li M., Chen Y., Li G. (2020). A review of computer vision technologies for plant phenotyping. Comput. Electron. Agric..

[10] Moreno R., Mayer R. (2007). Interactive multimodal learning environments: Special issue on interactive learning environments: Contemporary issues and trends. Educ. Psychol. Rev..

[11] Xu C., Xie W. (2017). Thinking on Urban Landscape Installation Design Based on Entity Interaction Technology. Art Technol..

[12] Cooper A., Reimann R., Cronin D., Liu S. (2008). About Face 3.0 Essence of Interactive Design.

[13] Card K.S., Moran T.P., Newell A. (1983). The Psychology of Human-Computer Interaction.

[14] Ishii H., Ullmer B. Tangible Bits: Towards Seamless Interfaces between People, Bits and Atoms. Proceedings of the ACM SIGCHI Conference on Human Factors in Computing Systems.

[15] Zuckerman O., Gal-Oz A. (2014). Deconstructing gamification: Evaluating the effectiveness of continuous measurement, virtual rewards, and social comparison for promoting physical activity. Pers. Ubiquitous Comput..

[16] Zuckerman O., Gal-Oz A. (2013). To TUl or not to TUI: Evaluating performance and preference in tangible vs. graphical user interfaces. Int. J. Hum. Comput. Stud..

[17] Stolterman E., Wiberg M. (2010). Concept-driven interaction design research. Hum.–Comput. Interact..

[18] Zhang M. (2011). “Human landscape interaction” in landscape design in the new era. Value Eng..

[19] Yu F., Tang J., Yin W., Sun Y., Tian H., Wu H., Wang H. (2020). ERNIE-ViL: Knowledge Enhanced Vision-Language Representations through Scene Graph. arXiv.

[20] Venturelli S., Paula Barretto F. (2015). BioCyberUrban ParQ: Brasilia’s Smart National Park as an Extension of Our Senses. Proceedings of the International Conference on Universal Access in Human-Computer Interaction.

[21] Forman R.T.T. (2014). Urban Ecology: Science of Cities.

[22] Cooper A., Reimann R. (2005). About Face 2.0 Software Conceptual Revolution: The Essence of Interactive Design.

[23] Turk M., Hua G. (2013). Vision-based interaction. Synth. Lect. Comput. Vis..

[24] Jia Y. (2020). Constructing Virtual Reality Exhibitions with Multimodal Interactions.

[25] Davies E.R. (2004). Machine Vision: Theory, Algorithms, Practicalities.

[26] Hammerschmidt J., Hermann T., Walender A., Krömker N. InfoPlant: Multimodal augmentation of plants for enhanced human-computer interaction. Proceedings of the 2015 6th IEEE International Conference on Cognitive Infocommunications (CogInfoCom), IEEE.

[27] Bianchi-Berthouze N., Lisetti C.L. (2002). Modeling multimodal expression of user’s affective subjective experience. User Model. User-Adapt. Interact..

[28] Payne S.J., Howes A. (2013). Adaptive interaction: A utility maximization approach to understanding human interaction with technology. Synth. Lect. Hum. Cent. Inform..

[29] Bernasco W., Hoeben E.M., Koelma D., Liebst L.S., Thomas J., Appelman J., Snoek C.G.M., Lindegaard M.R. (2021). Promise into practice: Application of computer vision in empirical research on social distancing. Sociol. Methods Res..

[30] Wang Y., Towara T., Anderl R. (2017). Technology Landscape 4.0. Proceedings of the World Congress on Engineering and Computer Science.

[31] Clouston B., Zixin C., Ci’an X. (1992). Landscape Garden Plant Configuration.

[32] Cooper A., Reimann R., Cronin D. (2014). About Face: The Essentials of Interaction Design.

[33] Le H.A., Mensink T., Das P., Karaoglu S., Gevers T. Eden: Multimodal synthetic dataset of enclosed garden scenes. Proceedings of the IEEE/CVF Winter Conference on Applications of Computer Vision.

[34] Booth N.K. (1989). Basic Elements of Landscape Architectural Design.

[35] Guo L. (2020). Research on Interactive Product Design for Plant Science. Master’s Theses.

[36] Cui J., Xu K.S., Gao J.F. (2005). Interactive virtual plant structure modeling based on L-system. J. Wuhan Univ. Technol. Transp. Sci. Eng. Ed..

[37] Portman M.E., Natapov A., Fisher-Gewirtzman D. (2015). To go where no man has gone before: Virtual reality in architecture, landscape architecture and environmental planning. Comput. Environ. Urban Syst..

[38] Engler M. (2015). Cut and Paste Urban Landscape: The Work of Gordon Cullen.

[39] Chen X., Bishop I.D., Hamid A.R.A. Community exploration of changing landscape values: The role of the virtual environment. Proceedings of the Digital Image Computing-Techniques and Applications.

[40] Orland B., Budthimedhee K., Uusitalo J. (2001). Considering virtual worlds as representations of landscape realities and as tools for landscape planning. Landsc. Urban Plan..

[41] Sheppard S.R.J., Salter J.D. (2004). Landscape and planning|The role of visualization in Forest Planning. Encyclopedia of Forest Sciences.

[42] Pettit C., Sheth F., Harvey W., Cox M. Building a 3D object library for visualising landscape futures. Proceedings of the 18th World IMACS Congress and MODSIM09 International Congress on Modelling and Simulation.

[43] Zhang Z. (2008). Growth Model and Visualization Based on Plant Interactions.

[44] Leitao A.B., Ahern J. (2002). Applying landscape ecological concepts and metrics in sustainable landscape planning. Landsc. Urban Plan..

[45] Wilmes S.E.D., Siry C. (2021). Multimodal interaction analysis: A powerful tool for examining plurilingual students’ engagement in science practices. Res. Sci. Educ..

[46] Van Dam A., Laidlaw D.H., Simpson R.M. (2002). Experiments in immersive virtual reality for scientific visualization. Comput. Graph..

[47] Haney D. (2010). When Modern Was Green: Life and Work of Landscape Architect Leberecht Migge.

[48] Ahern J. (2013). Urban landscape sustainability and resilience: The promise and challenges of integrating ecology with urban planning and design. Landsc. Ecol..

[49] Hellmund P.C., Smith D. (2013). Designing Greenways: Sustainable Landscapes for Nature and People.

